# Development and multimodal validation of a substance misuse algorithm for referral to treatment using artificial intelligence (SMART-AI): a retrospective deep learning study

**DOI:** 10.1016/S2589-7500(22)00041-3

**Published:** 2022-06

**Authors:** Majid Afshar, Brihat Sharma, Dmitriy Dligach, Madeline Oguss, Randall Brown, Neeraj Chhabra, Hale M Thompson, Talar Markossian, Cara Joyce, Matthew M Churpek, Niranjan S Karnik

**Affiliations:** Department of Medicine (M Afshar MD, M Oguss MPH, M M Churpek MD) and Department of Family Medicine (Prof R Brown MD), School of Medicine and Public Health, University of Wisconsin, Madison, WI, USA; Department of Psychiatry and Behavioral Sciences, Rush University Medical Center, Chicago, IL, USA (B Sharma MS, H M Thompson PhD, Prof N S Karnik MD); Department of Computer Science (D Dligach PhD), Parkinson School of Health Sciences and Public Health (T Markossian PhD), and Clinical Research Office, Stritch School of Medicine (C Joyce PhD), Loyola University Chicago, Chicago, IL, USA; Department of Emergency Medicine, Cook County Health, Chicago, IL, USA (N Chhabra MD)

## Abstract

**Background:**

Substance misuse is a heterogeneous and complex set of behavioural conditions that are highly prevalent in hospital settings and frequently co-occur. Few hospital-wide solutions exist to comprehensively and reliably identify these conditions to prioritise care and guide treatment. The aim of this study was to apply natural language processing (NLP) to clinical notes collected in the electronic health record (EHR) to accurately screen for substance misuse.

**Methods:**

The model was trained and developed on a reference dataset derived from a hospital-wide programme at Rush University Medical Center (RUMC), Chicago, IL, USA, that used structured diagnostic interviews to manually screen admitted patients over 27 months (between Oct 1, 2017, and Dec 31, 2019; n=54 915). The Alcohol Use Disorder Identification Test and Drug Abuse Screening Tool served as reference standards. The first 24 h of notes in the EHR were mapped to standardised medical vocabulary and fed into single-label, multilabel, and multilabel with auxillary-task neural network models. Temporal validation of the model was done using data from the subsequent 12 months on a subset of RUMC patients (n=16 917). External validation was done using data from Loyola University Medical Center, Chicago, IL, USA between Jan 1, 2007, and Sept 30, 2017 (n=1991 adult patients). The primary outcome was discrimination for alcohol misuse, opioid misuse, or non-opioid drug misuse. Discrimination was assessed by the area under the receiver operating characteristic curve (AUROC). Calibration slope and intercept were measured with the unreliability index. Bias assessments were performed across demographic subgroups.

**Findings:**

The model was trained on a cohort that had 3·5% misuse (n=1 921) with any type of substance. 220 (11%) of 1921 patients with substance misuse had more than one type of misuse. The multilabel convolutional neural network classifier had a mean AUROC of 0·97 (95% CI 0·96–0·98) during temporal validation for all types of substance misuse. The model was well calibrated and showed good face validity with model features containing explicit mentions of aberrant drug-taking behaviour. A false-negative rate of 0·18–0·19 and a false-positive rate of 0·03 between non-Hispanic Black and non-Hispanic White groups occurred. In external validation, the AUROCs for alcohol and opioid misuse were 0·88 (95% CI 0·86–0·90) and 0·94 (0·92–0·95), respectively.

**Interpretation:**

We developed a novel and accurate approach to leveraging the first 24 h of EHR notes for screening multiple types of substance misuse.

**Funding:**

National Institute On Drug Abuse, National Institutes of Health.

## Introduction

Overdose deaths from opioid misuse have reached an all-time high during the COVID-19 pandemic, and rates of alcohol withdrawal in hospitalised patients have also increased.^1,2^ The number of substance-use-related hospital visits in the USA continues to grow and now outpaces visits for heart disease and respiratory failure.^3^ Yet, hospital screening rates for substance misuse remain low, despite recommendations to implement screening tools.^4^ The recommendation from the US Preventive Services Task Force for Unhealthy Drug Use Screening is to incorporate interviewer-administered or self-administered tools.^5^ Screening questionnaires can be built into electronic health record (EHR) notes, but they require additional staff and time. Few staff administer questionnaires and patients might not be able to self-report; thus, screening adherence remains low or nonexistent within many health systems—automated solutions could breach the gap.

An automated tool that runs off the notes collected during usual care could enable full screening effort on all hospitalised patients. Information about substance use in providers’ intake notes is routinely collected but it is neither organised nor prioritised for clinical decision support. Moreover, the free text format is another challenge in building data-driven approaches. Automated, data-driven solutions with natural language processing (NLP) can analyse the free text and extract semantics, capturing important features from the unstructured format of clinical notes that can be fed into machine learning classifiers for computable phenotyping.^6^ A comprehensive and automated approach to substance misuse screening using methods in NLP could offer key opportunities to augment clinical workflow and improve care.

The goal of our study is to build an automated NLP tool for screening substance misuse using data collected in the EHR notes during usual care and to show its screening capabilities. Our previous work focused on single use classifiers.^7,8^ In this study, we examine the development of one classifier to screen for multiple types of substance misuse for ease of implementation, training it with co-learning across labels (polysubstance use), and efficiency in having one model. More specifically, we aim to develop and validate a machine learning classifier that can identify alcohol misuse, opioid misuse, and non-opioid drug misuse using EHR notes collected during the first day of hospitalisation. Current recommendations to screen for multiple types of substance misuse can be combined into one machine learning model to reduce redundancy in clinical decision support to the provider and potentially reduce alarm fatigue.

## Methods

### Study design and population

**Figure 1:**
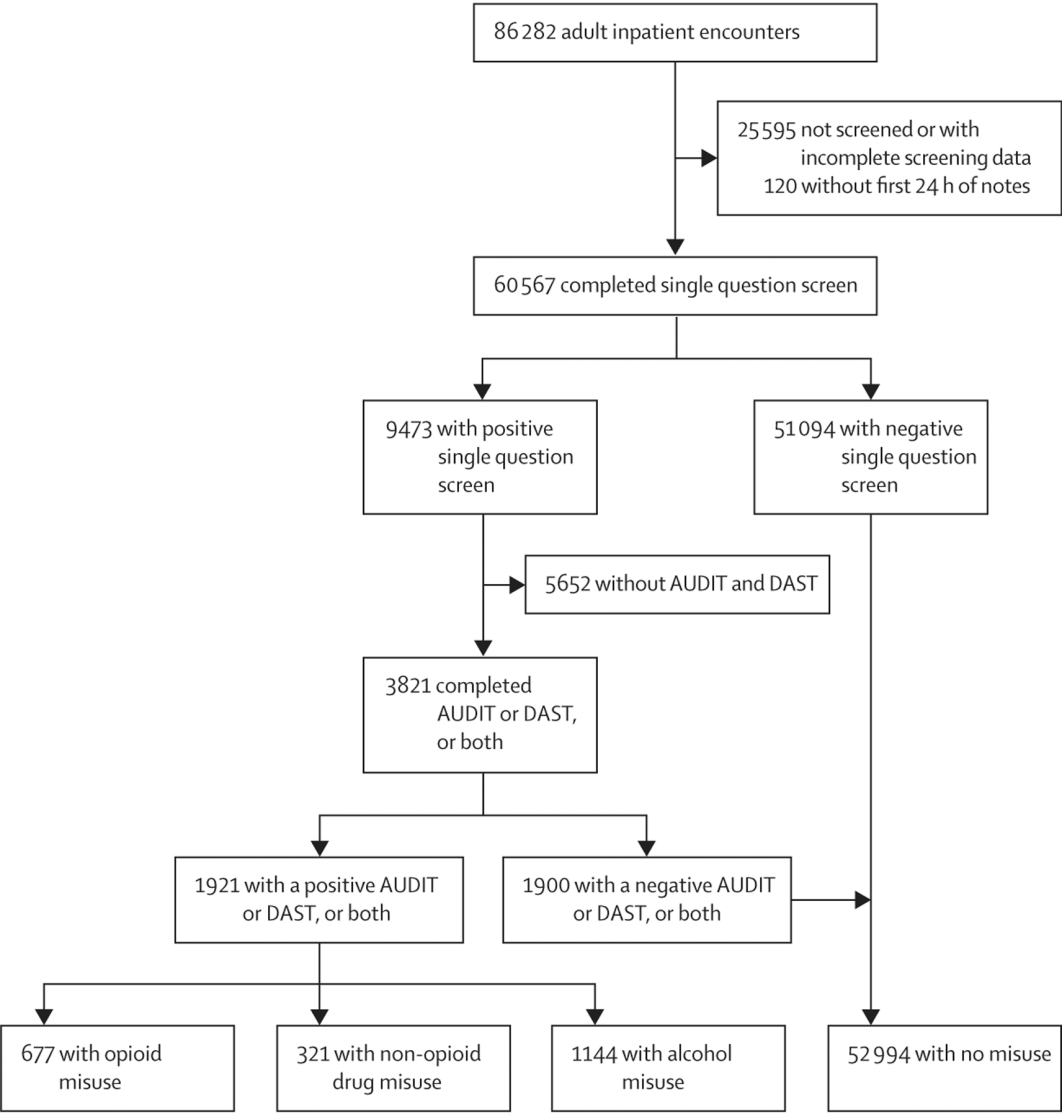
Patient flow diagram for training cohort AUDIT=Alcohol Use Disorders Identification Test. DAST=Drug Abuse Screening Test. Positive DAST are scores of 2 or higher for both sexes and positive AUDIT are scores of 5 or higher for women and 8 or higher for men. More than one type of substance misuse might apply to the same individual.

Models were trained and developed using the first 24 h of EHR notes from a cohort of hospitalised adult patients (aged 18 years and older) screened for substance misuse at Rush University Medical Center (RUMC), Chicago, IL, USA, between Oct 1, 2017, and Dec 31, 2019 (figure 1). Temporal validation was done on the cohort of patients during the subsequent 12 months in 2020. External validation was done on a sampling of 1991 adult patients hospitalised at Loyola University Medical Center (LUMC) Chicago, IL, USA, between Jan 1, 2007 and Sept 30, 2017. Inpatient demographics and hospital characteristics for RUMC and LUMC are detailed in the appendix (p 1). The primary analysis is focused on the temporal validation cohort at RUMC to support future deployment of the tool and examine its effect on outcomes and treatment referrals in a prospective study.

### Reference dataset of hospitalised patients with substance misuse

Cases (defined as someone positive for misuse) and non-cases at RUMC were determined using previously validated interview-administered questionnaires delivered as part of a hospital-wide screening programme.^5,9,10^ The screening questionnaires were not designed to make a diagnosis of a substance use disorder according to the Diagnostic and Statistical Manual of Mental Disorders. Rather, the questionnaires were to identify unhealthy behaviours that might be a risk for developing a substance use disorder. Patients first received a universal screening with a single questionnaire for alcohol and drugs: “How many times in the past year have you had [X] or more drinks in a day?” and “How many times in the past year have you used an illegal drug or used a prescription medication for non-medical reasons?” Patients with positive universal questionnaires subsequently received the Alcohol Use Disorder Identification Test (AUDIT) or the Drug Abuse Screening Tool (DAST), or both. Sex-specific cutoff points for alcohol misuse (score of ≥5 for women and score of ≥8 for men) were used for cases from the AUDIT, whereas for drug misuse a score of >2 for both sexes was used for the DAST.^10,11^ Drugs were categorised into opioid misuse (heroin and non-medical opioids) and non-opioid drug misuse (cocaine, stimulants, sedative-hypnotic, etc; figure 1). Full details of the hospital-wide screening programme have been previously described.^12^ The hospital screening data were collected by the care team as scores and entered into EHR flowsheets as part of usual care and in accordance with best practices and recommendations for screening by the US Preventative Task Force. The manual screening data were not recorded in the notes and did not contaminate the study data used in model development.

For external validation, no formal screening programme was available at LUMC, so a random sampling of unique hospital encounters (defined as hospital visits or hospitalisations) was performed for the reference labels across two separate datasets that were chart reviewed and labelled with cases for alcohol misuse and for opioid misuse. Trained medical annotators reviewed the medical charts for the likelihood of alcohol misuse or opioid misuse. Inter-rater reliability of Cohen’s kappa coefficient of 0·75 or greater was reached with a critical care specialist and an addiction researcher (MA and NSK) before independent review. The sampling method and composition of the datasets have been previously described.^7^

### Natural language processing of clinical notes

The EHR system at RUMC is Epic (Epic Systems Corporation, Verona, WI, USA). Epic has many different instances, with customisations for each health system; however, standardisation of clinical notes across EHRs is not available. Therefore, we designed a pragmatic approach to collect notes from the first 24 h of hospitalisation for training data. In 2016, there were about 35·7 million US hospitalisations, with a mean length of stay of 4·6 days^13^—ample time for the classifier to operate and for subsequent consultations and treatment by an addiction provider to occur during the hospitalisation.

Linguistic processing of all clinical notes, collected between Oct 1, 2017, and Dec 31, 2019, was performed in the clinical Text and Knowledge Extraction System (version 4.0; cTAKES).^14^ cTAKES can recognise words or phrases from the notes as medical terms that represent domain concepts (named entities) derived from the Unified Medical Language System (UMLS). The spans of the UMLS Metathesaurus named entities (diseases, symptoms, anatomical sites, medications, and procedures) were mapped from the clinical notes and organised into standardised terms as concept-unique identifiers (CUIs).^14^ For example, the named entity mentioned for substance misuse is mapped to CUI C4540992 which is different than family history of substance misuse (C4540855). The CUIs were fed into machine learning models using two approaches: the-bagof-CUIs method, with a matrix of 37 317 CUIs, and implementation of an embedding layer with a range between 300 and 1024 dimensions.

### Model architectures

We examined two approaches to build a substance misuse algorithm for referral to treatment using artificial intelligence (SMART-AI): a multilabel and a multilabel with auxiliary task learning. A multilabel classifier allows for all types of substance misuse to be identified jointly with a binary label (yes or no) for each substance. An auxiliary task classifier allows for additional auxiliary labels to be added and could help the model better learn the primary labels for substance misuse (appendix p 4). The auxiliary labels were derived from the International Classification of Diseases (ICD)-10 codes for substance use and comorbid conditions (appendix pp 5–7). The models across the approaches include different deep learning neural networks that take the CUI data as inputs from the first 24 h of EHR notes for supervised learning. We examined multiple architectures, including logistic regression, feed-forward neural networks, convolutional neural networks, deep averaging networks, and transformer neural networks. A detailed description of the architectures and our random search approach for determining hyperparameters are detailed in the appendix (pp 8–11).

The first 27 months (between Oct 1, 2017, and Dec 31, 2019) of hospitalisations from RUMC (n=54 915) served as the dataset for model training and development. We used 90% (n=49 423) of the dataset for training and the remaining 10% (n=5492) for model selection. The evaluation metric used for model selection was the macro-averaged (the average from the individual metrics for each type of misuse) area under the precision-recall curve (AUPRC). AUPRC was chosen to account for the low prevalence of cases to minimise false negatives for screening and maximise positive predictive values to avoid potential stigma from false positives. The area under the receiver operating characteristic curve (AUROC) was also reported. Hospitalisations from the subsequent 12 months (between Jan 1, 2020, and Dec 31, 2020; n=16 917) served as temporal validation to represent the most recent medical practice behaviours. The data corpus from RUMC represents the full sample size of patients manually screened and the external validation cohort from LUMC represents a convenience sample of chart-reviewed patients. Developed models were also compared with single-substance-use classifiers that were previously published for alcohol and opioid misuse.^7,8^

### Analysis plan

Statistical tests to examine baseline characteristics were done using the chi-square test for proportions and Kruskal-Wallis nonparametric tests for quantitative variables. The primary outcome was discrimination for alcohol misuse, opioid misuse, or non-opioid drug misuse as measured by the macro-averaged AUPRC and AUROC. Analysis was done at the hospital encounter level since screening was performed independently for each hospitalisation. To assess the model’s performance for decision-making, results were also shown across risk thresholds as classification plots.^15^ Multiple cutoff points were examined for optimal threshold selection, including the point on the AUROC curve that minimised the difference between sensitivity and specificity, and Youden’s J statistic. The metrics—sensitivity, specificity, positive predictive value, and negative predictive value—were calculated at optimal cutoff points with 95% CIs.

Calibration slope and calibration intercept were reported with an unreliability index for the accuracy of absolute risk estimates across deciles of predicted probabilities from the classifier with the best AUROC results.^16,17^ The temporal validation cohort included the COVID-19 pandemic period. During this time period, hospitalisation characteristics changed and staffing for the addiction consult service temporarily changed from preceding years; therefore, recalibration with isotonic regression was applied to account for the change in the prevalence of cases.^18^

The number needed to evaluate, also known as the workup to detection ratio, was calculated as one divided by a positive predictive value.

### Bias assessment for model equity

In a secondary analysis, we assessed for bias in the temporal validation cohort by age, sex, and race and ethnicity groups. Age was divided into two groups a priori based on US census age groups. Race and ethnicity were self-reported into the EHR and categorised into the following four groups: (1) non-Hispanic Black; (2) non-Hispanic White; (3) Hispanic; and (4) mixed. Due to small sample sizes, the mixed race and ethnicity group included the Asian, Native-American or Alaskan-Native, Native-Hawaiian or other Pacific-Islander, other race and ethnicity, and answer refused or unknown groups. Bias assessment metrics were previously described^19^ and included false-discovery rate, false-positive rate, false-omission rate, and false-negative rate with 95% CIs.

### eXplainable artificial intelligence for face validity

Local interpretable model-agnostic explanations (LIME) is a visualisation technique that helps explain individual predictions in a deep learning model.^20^ As the LIME is model agnostic it follows the assumption that every complex model can be simplified to a linear model at the individual level. The simple model was ran on a single instance (eg, a hospital encounter) from the full sample size and used to explain the predictions of the more complex model locally. A total of 2000 randomly selected hospital encounters from the training data were examined. Model fit was reported by gathering the median *R*^2^ for variance explained across the 2000 predictions. Local feature weights as beta coefficients were used to interpret which CUIs contributed most to the model’s predictions, and the weights were averaged across the 2000 predictions and ranked for each substance misuse type. The full method for LIME is detailed in the appendix (p 11). This study was approved by the institutional review board at RUMC and LUMC and all analyses were done at the site where the data originated. The study was determined to meet the criteria for exempt human participants research at both institutions, thus patient consent was not required. Analysis was done using Python (version 3.6.5) and R Studio (version 1.1.463). The study followed the Transparent Reporting of Multivariable Prediction Model for Individual Prognosis or Diagnosis (TRIPOD) reporting guidelines^21^ (appendix pp 2–3).

### Role of the funding source

The funders had no role in the study design; collection, analysis, and interpretation of data; writing of the report; or the decision to submit for publication.

## Results

Between Oct 1, 2017, and Dec 31, 2019, 60 567 (70%) of 86 282 hospitalised patients completed at least one single-question manual screening for substance misuse. A comparison between the screened cohort and the cohort without screening are detailed in the appendix (pp 12–13). The cohort without screening had greater median age, greater proportion of in-hospital deaths, and a greater proportion of patients discharged against medical advice. The final model was trained on the screened cohort of 54 915 adult patients with available clinical notes. Results from manual screening identified that 1921 (3·5%) of 54 915 petients had any type of substance misuse. The demographics and patient characteristics of the final training cohort are detailed in the appendix (pp 14–15).

The training data from the first 24 h of hospitalisation was composed of 664 836 notes and 81 726 193 CUIs with a vocabulary of 37 317 unique CUI terms. The most frequent note types included in the analysis were: progress notes (n=143 112), emergency department notes (n=121 129), and medical history and physical notes (n=56 264).

The 2020 temporal validation dataset had 16 917 (54%) of 31 328 hospitalisations manually screened; 1023 (6%) of 16 917 had any type of substance misuse. 112 (11%) of 1023 patients with any type of substance misuse had more than one type of substance misuse (table 1). In comparison with patients without substance misuse, patients with any substance misuse had a lower median age, a greater proportion of them had mental health conditions; patients with opioid misuse had the greatest proportion that left the hospital against medical advice.

Among the candidate model architectures, the multilabel convolutional neural network model provided the best predictive metrics (table 2). The addition of auxiliary labels had similar results to the multilabel model in temporal and external validation. The convolutional neural network model without auxiliary labels in the temporal validation dataset had a mean AUROC of 0·97 (95% CI 0·96–0·98) and AUPRC of 0·69 (95% CI 0·64–0·74) for the different types of substance misuse. This model discriminated better for alcohol misuse and opioid misuse than for non-opioid drug misuse. In external validation, the AUPRC and AUROC for alcohol and opioid misuse were both above 0·85 (table 2). The multilabel convolutional neural network had a higher AUROC and AUPRC than the single-label alcohol misuse classifier and the single-label opioid misuse classifier (table 2).

During temporal validation, the optimal cutoff point for each type of substance misuse was 0·05 after recalibration. At that cutoff point, prediction of alcohol misuse had a sensitivity of 0·77 (95% CI 0·73–0·80), specificity of 0·99 (0·99–0·99), positive predictive value of 0·68 (0·64–0·71), and negative predictive value of 0·99 (0·99–0·99). For opioid misuse, the sensitivity was 0·87 (0·84–0·90), specificity 0·99 (0·99–0·99), positive predictive value 0·76 (0·72–0·88), and negative predictive value 0·99 (0·99–0·99). For non-opioid drug misuse, the sensitivity was 0·60 (0·52–0·68), specificity 0·99 (0·99–0·99), positive predictive value 0·39 (0·33–0·45), and negative predictive value 0·99 (0·99–0·99). A range of the uncalibrated cutoff points with their confusion matrix are listed in the appendix (pp 16–17) from the temporal and external validation cohorts.

**Figure 2:**
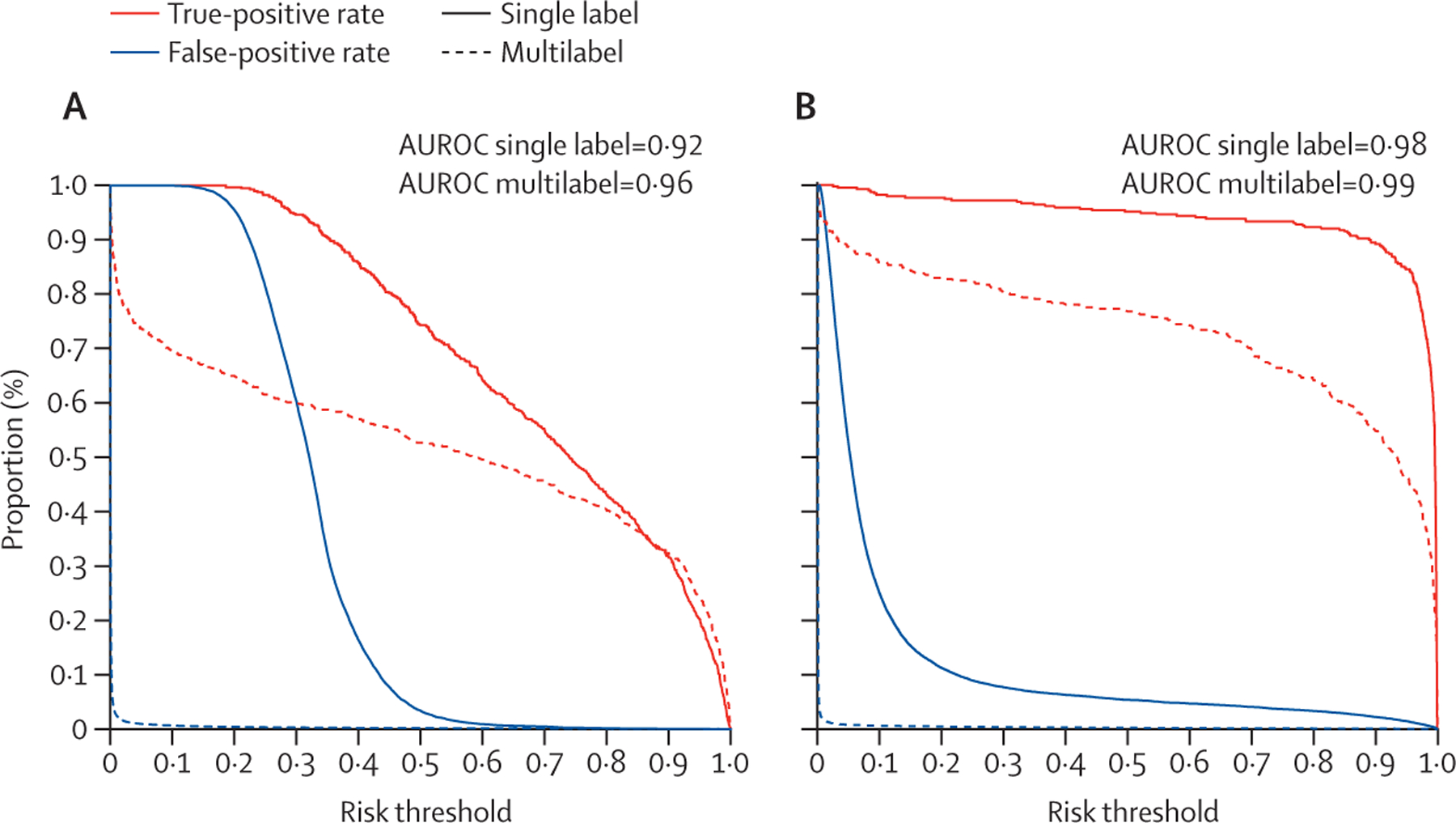
Classification plots for temporal validation comparing single-label alcohol (A) and single-label opioid (B) classifiers to multilabel convolutional neural network classifier (A) Single-label logistic regression alcohol misuse classifier versus multilabel convolutional neural network alcohol misuse classifier. (B) Single-label convolutional neural network opioid misuse classifier versus multilabel convolutional neural network opioid misuse classifier. AUCROC=area under the receiver operating characteristic curve.

Classification plots showed lower false-positive rates across most thresholds when compared with the single-alcohol and single-opioid classifiers (figure 2A, B). The number of patients needed to evaluate was 1·5 for alcohol misuse, 1·3 for opioid misuse, and 2·6 for non-opioid drug misuse. This would create 39, 26, and 16 alerts per 1000 patients for each group, respectively. After recalibration, the calibration intercepts were 0·06 (−0·10 to 0·23) for alcohol misuse, 0·08 (−0·14 to 0·29) for opioid misuse, and 0·05 (−0·20 to 0·30) for non-opioid drug misuse. The calibration slopes were 0·97 (0·92 to 1·03) for alcohol misuse, 0·94 (0·87 to 1·02) for opioid misuse, and 0·94 (0·86 to 1·02) for non-opioid drug misuse. The unreliability index showed the classifier was well calibrated (p<0·05; appendix pp 18–20).

Of the 1091 patients identified by the multilabel convolutional neural network classifier, 982 (90%) also had an ICD code for substance misuse during hospitalisation—a proportion greater than that identified by manual screening (886 [86%] of 1024 identified by manual screening had an ICD code for substance misuse). Furthermore, 115 (24%) of the 479 patients identified by the multilabel convolutional neural network classifier for opioid misuse had an ICD code for opioid or drug overdose or intoxication, or both. Of the patients that did not receive a manual screening during the temporal validation period, the convolutional neural network multilabel classifier would have identified another 959 patients.

The global LIME results had a median *R*^2^ range of 0·95 to 0·96 across the substance misuse types. The classifier showed good face validity with top features across the types of substance misuse having explicit mentions of aberrant drug-taking behaviour. The top features were ethanol for alcohol misuse, heroin for opioid misuse, and cocaine for non-opioid drug misuse. The top 25 features positive for AI explainability are listed in the appendix (p 21) for each substance misuse type.

In bias assessments of the SMART-AI the false-omission rate and the false-positive rate remained low at 3% or less across subgroups of age, sex, and race and ethnicity (table 3). The false-negative rates and false-positive rates were similar between non-Hispanic Black and non-Hispanic White groups. The false-discovery rate was higher in non-Hispanic Black individuals (0·29 [95% CI 0·26–0·34]) than in non-Hispanic White individuals (0·22 [0·18–0·26]). The false-discovery rate was higher for those aged 45 years or older compared with those aged 18–44 years (0·30 [0·26–0·33] *vs* 0·19 [0·16–0·23]).

## Discussion

We developed and then temporally and externally validated an accurate screening tool to identify cases of substance misuse. Our model showed good calibration and face validity, with few disparities across patient subgroups during temporal validation in the COVID-19 pandemic year (Jan 1–Dec 31, 2020). With this model we offer an automated approach for screening multiple types of substance misuse simultaneously.

Prediction performance can change over time or in different patient settings, where prevalence of substance misuse varies. We showed changes in prevalence over time (from 70% to 54%) during the COVID-19 pandemic, which required the classifier to be recalibrated so that predicted risk would not be underestimated across individuals. Calibration is a major reason for failure in models to perform well in external settings and capture appropriate risk among groups.^21^ Continually redeveloping a new model is not feasible because it is time consuming, requires abundant data, and wastes potentially useful information from existing models.^22^ Thus, model updating or recalibration is an efficient alternative and recommended by the TRIPOD guidelines.^21^ Our updated machine learning classifier had an acceptable alarm rate and number needed to evaluate the alerts. These characteristics support an automated screening that could potentially overcome staffing challenges and improve hospital screening rates.

Substance misuse is a complex condition that frequently occurs as polysubstance misuse. Our model offers the advantage of modelling different types of substance use jointly rather than using individual models. Machine learning methods have an advantage over traditional statistical and textual approaches, because they can examine the complexities of all interactions and combinations of terms,^23^ as illustrated by our convolutional neural network model, which had more than 12 million parameters from a vocabulary of more than 35 000 unique terms (appendix p 6). Our multilabel classifier outperformed the single-label opioid and single-label alcohol classifiers, with high sensitivity, including that of 87% for opioid misuse.

A machine learning approach treats each word as a feature and does not require subject matter expertise, unlike models that relied on keyword, rule-based systems.^24^ Other AI-based systems were trained on data that were not readily available in the EHR, focused on a subset of patients with opioid use disorders, or were limited to structured data.^25,26^ Our approach incorporated EHR notes and their interpretability as features in our deep learning models with approaches in eXplainable artificial intelligence, and showed that the features for identifying substance misuse were relevant terms and discovery was not limited to just explicit terms of substance use.

The classifier had lower performance metrics for the non-opioid drug misuse group. This is probably due to the small sample size and heterogeneity in types of drug use. However, our classifier provides the flexibility to turn off for any single label. This flexibility might be necessary for the non-opioid group, as treatment options remain scarce.^27^ Treatments for cocaine, methamphetamines, or benzodiazepines are less established than for alcohol or opioid misuse but remain important to monitor.

Currently, the existing hospital-wide screening at RUMC is part of a larger screening, brief intervention, and referral to treatment programme. With a very low false-negative rate, SMART-AI could replace the existing universal screening in order to target patients for a follow-up confirmatory screening with risk stratification. SMART-AI is not intended to replace decision-making by a physician, and a confirmatory screen is still needed to reduce the false positives and potential stigma from misclassification.

Our model showed good estimates of absolute risk across deciles of predicted probabilities with a calibration intercept that approximated 0 and calibration slope that approximated 1. We did not identify major disparities across subgroups for type I (false-positive rate) and type II (false-negative rate) errors. For screening purposes, the false-negative rate is the focus and here remained low across subgroups, without major disparities, suggesting few cases would be missed in a hospital-wide programme. The false-positive rate was minor across subgroups, and reduced potential stigma in mislabelling individuals with substance misuse. Although false-positive and false-negative rates were low, they still exist and will require disclosure and education to hospital operations and quality stakeholders before deployment of hospital-wide screening. The disparity was in the false-discovery rate for non-Hispanic Black patients, as well as older age groups, and might indicate that the system overestimates risk in this group. Post-hoc mitigation methods in the opioid misuse classifier were previously shown to improve disparities and could be considered before deployment.^28^ Close observation is still required during deployment to identify unintended consequences. Implicit bias in provider notes remains a real problem in treatment of patients, especially in patients with substance misuse. More work is needed to examine the quality of notes and surfacing biases. Next step before deployment of our screening tool is a prospective clinical study with an oversight committee and data safety monitoring board tasked to examine disparities in performance and patient selection.

Our study has several limitations. Although we showed good external validation at a suburban medical centre, our classifier should be tested in rural regions. Chicago has long encountered an opioid epidemic with heroin use in middle-aged non-Hispanic Black people,^29^ so the model was trained with a greater proportion of these individuals than encountered within other health systems. As drug markets evolve, additional model updates might be needed to account for demographic and consumption changes. The rates of substance use in our study were low and might represent under-reporting. We did attempt to look at multiple architectures spanning different types of neural networks and feature engineering, but our approach was not exhaustive and other architectures (eg, bidirectional encoder representations from transformers) have shown promise in classification tasks; however, our model was trained on CUIs and not raw text.^30^ Moreover, we show an adequate criterion and face validity of our classifier, but its effect on programme fidelity and health outcomes remains unknown. Patients needing treatment for their substance misuse are restricted by the availability and cost of treatment services. Although many health systems might improve screening with automated tools, a gap analysis for treatment services and capacity are still needed, as are efforts to reduce stigma. Future research is needed to address feasibility in deployment of our classifier, patient and staff acceptance of screening, and ethical concerns such as perpetuating stigmatising language before large-scale implementation.

In conclusion, our results showed that clinical notes from hospitalisation can be used to identify substance misuse accurately with the help of AI to potentially improve screening rates.

## Supplementary Material

1

## Figures and Tables

**Table 1:** Patient characteristics and outcomes from temporal validation cohort (n=16 917)

	Alcohol misuse only (n=466)	Opioid misuse only (n=341)	Non-opioid misuse only (n=104)	Polysubstance misuse (n=112)	No misuse (n=15 894)
Age, years	48·0 (38·3–57·8)	52·0 (38·0–60·0)	53·0 (36·8–59·0)	38·0 (32·0–53·0)	59·0 (39·0–70·0)
Sex					
Male	324 (69·5%)	228 (66·9%)	70 (67·3%)	86 (76·8%)	6265 (39·4%)
Female	142 (30·5%)	113 (33·1%),	34 (32·7%)	26 (23·2%)	9629 (60·6%)
Race and ethnicity					
Non-Hispanic White	185 (39·7%)	115 (33·7%)	19 (18·3%)	34 (30·4%)	5803 (36·5%)
Non-Hispanic Black	155 (33·3%)	183 (53·7%)	65 (62·5%)	42 (37·5%)	5745 (36·1%)
Hispanic	99 (21·2%)	22 (6·5%)	15 (14·4%)	23 (20·5%)	3170 (19·9%)
Mixed*	27 (5·8%)	21 (6·2%)	5 (4·8%)	13 (11·6%)	1176 (7·3%)
AUDIT score	22·5 (14·0–29·0; n=466)	1·0 (0·0–2·3; n=44)	3·0 (1·0–4·0; n=20)	23·5 (16·0–31·0; n=112)	2·0 (1·0–4·0; n=598)
DAST score	1·0 (0·75–2·0; n=71)	7·0 (5·0–8·0; n=341)	4·0 (2·0–5·0; n=104)	6·0 (4·0–8·0; n=112)	1·0 (0·0–1·0; n=416)
Insurance					
Medicare	255 (54·7%)	232 (68·0%)	20 (19·2%)	8 (7·1%)	5553 (34·9%)
Medicaid	63 (13·5%)	41 (12·0%)	63 (60·6%)	72 (64·3%)	5720 (35·9%)
Private	99 (21·2%)	52 (15·2%)	16 (15·4%)	26 (23·2%)	4084 (25·7%)
Other	49 (10·5%)	16 (4·7%)	5 (4·8%)	6 (5·4%)	537 (3·4%)
Comorbidities					
Hypertension	243 (52·1%)	167 (48·9%)	65 (62·5%)	39 (34·8%)	9545 (60·1%)
Renal failure	30 (6·4%)	56 (16·4%)	19 (18·3%)	4 (3·6%)	3571 (22·5%)
Neurologic	104 (22·3%)	53 (15·5%)	14 (13·5%)	14 (12·5%)	2569 (16·2%)
Congestive heart failure	39 (8·4%)	64 (18·8%)	28 (26·9%)	6 (5·4%)	2883 (18·1%)
Diabetes	83 (17·8%)	60 (17·6%)	30 (28·8%)	16 (14·3%)	4767 (29·9%)
Liver disease	168 (36·1%)	43 (12·6%)	8 (7·7%)	12 (10·7%)	1258 (7·9%)
Chronic lung disease	75 (16·1%)	132 (38·7%)	40 (38·5%)	22 (19·6%)	3431 (21·6%)
Psychiatric disorders	80 (17·2%)	65 (19·1%)	44 (42·3%)	45 (40·2%)	904 (5·7%)
Depression	153 (32·8%)	93 (27·3%)	27 (25·9%)	45 (40·2%)	2680 (16·9%)
Alcohol misuse	387 (83·0%)	25 (7·3%)	27 (25·9%)	91 (81·3%)	399 (2·5%)
Drug misuse	66 (14·2%)	326 (95·6%)	79 (75·9%)	85 (75·9%)	467 (2·9%)
AIDS	7 (1·5%)	13 (3·8%)	4 (3·8%)	0	103 (<1%)
Disposition					
Home	336 (72·1%)	195 (57·2%)	67 (64·4%)	85 (75·9%)	9549 (60·1%)
Death	2 (<1%)	5 (1·5%)	0	0	190 (1·2%)
Long-term residential care or short-term post-acute care	43 (9·2%)	47 (13·8%)	14 (13·4%)	10 (8·9%)	129 (<1%)
Against medical advice	17 (3·6%)	36 (10·6%)	1 (<1%)	4 (3·6%)	1417 (8·9%)
Other	68 (14·6%)	47 (13·8%)	22 (21·2%)	13 (11·6%)	4609 (28·9%)

Data are n (%) or median (IQR). Comparisons across all variables were significant with p values <0·01. Polysubstance misuse can include patients with alcohol, or opioid misuse, or non-opioid drug misuse, or any combination of the three.

* Mixed=Asian, Native American or Alaskan Native, Native Hawaiian or other Pacific Islander, other, or refused to answer or answer unknown.

**Table 2:** Full experiment models and results

	Temporal validation		External validation	
	AUPRC (95% CI)	AUROC (95% CI)	AUPRC (95% CI)	AUROC (95% CI)
**Baseline single classifiers**				
Alcohol*	0·70 (0·66–0·73)	0·92 (0·90–0·93)	NA	NA
Opioid†	0·75 (0·71–0·80)	0·98 (0·98–0·99)	NA	NA
**Multilabel logistic regression**				
Overall	0·65 (0·60–0·70)	0·96 (0·94–0·97)	NA	NA
Alcohol	0·73 (0·70–0·77)	0·95 (0·94–0·96)	0·92 (0·90–0·94)	0·89 (0·87–0·91)
Opioid	0·84 (0·80–0·87)	0·99 (0·98–0·99)	0·88 (0·85–0·91)	0·90 (0·88–0·92)
Non-opioid	0·39 (0·31–0·48)	0·94 (0·91–0·96)	NA	NA
**Multilabel deep averaging network**				
Overall	0·61 (0·56–0·66)	0·94 (0·92–0·95)	NA	NA
Alcohol	0·72 (0·68–0·76)	0·93 (0·91–0·94)	0·87 (0·84–0·89)	0·79 (0·76–0·82)
Opioid	0·82 (0·78–0·85)	0·98 (0·97–0·99)	0·75 (0·71–0·79)	0·78 (0·75–0·82)
Non-opioid	0·29 (0·22–0·37)	0·91 (0·88–0·93)	NA	NA
**Multilabel bag of CUIs**				
Overall	0·59 (0·55–0·64)	0·95 (0·94–0·96)	NA	NA
Alcohol	0·72 (0·68–0·76)	0·94 (0·93–0·95)	0·87 (0·85–0·90)	0·83 (0·80–0·85)
Opioid	0·83 (0·80–0·86)	0·99 (0·98–0·99)	0·84 (0·81–0·88)	0·88 (0·86–0·91)
Non-opioid	0·22 (0·17–0·29)	0·93 (0·91–0·95)	NA	NA
**Multilabel transformer**				
Overall	0·66 (0·61–0·71)	0·97 (0·96–0·97)	NA	NA
Alcohol	0·74 (0·70–0·77)	0·95 (0·94–0·96)	0·91 (0·89–0·93)	0·88 (0·85–0·90)
Opioid	0·84 (0·80–0·88)	0·99 (0·98–0·99)	0·87 (0·84–0·90)	0·90 (0·88–0·92)
Non-opioid	0·40 (0·32–0·49)	0·96 (0·95–0·97)	NA	NA
**Multilabel convolutional neural network**				
Overall	0·69 (0·64–0·74)	0·97 (0·96–0·98)	NA	NA
Alcohol	0·78 (0·75–0·82)	0·96 (0·95–0·97)	0·92 (0·90–0·93)	0·88 (0·86–0·90)
Opioid	0·87 (0·84–0·91)	0·99 (0·99–0·99)	0·91 (0·88–0·93)	0·94 (0·92–0·95)
Non-opioid	0·41 (0·34–0·50)	0·96 (0·94–0·98)	NA	NA
**Auxiliary task multilabel deep averaging network**				
Overall	0·64 (0·59–0·69)	0·94 (0·93–0·97)	NA	NA
Alcohol	0·70 (0·66–0·74)	0·93 (0·92–0·94)	0·83 (0·80–0·86)	0·78 (0·75–0·80)
Opioid	0·83 (0·79–0·86)	0·98 (0·97–0·98)	0·82 (0·78–0·85)	0·85 (0·82–0·88)
Non-opioid	0·40 (0·32–0·48)	0·92 (0·90–0·95)	NA	NA
**Auxiliary task multilabel bag of CUIs**				
Overall	0·58 (0·54–0·63)	0·95 (0·94–0·96)	NA	NA
Alcohol	0·71 (0·67–0·74)	0·94 (0·92–0·95)	0·87 (0·84–0·90)	0·83 (0·81–0·86)
Opioid	0·82 (0·79–0·86)	0·98 (0·98–0·99)	0·85 (0·81–0·88)	0·89 (0·86–0·91)
Non-opioid	0·22 (0·16–0·28)	0·94 (0·92–0·95)	NA	NA
**Auxiliary task multilabel transformer**				
Overall	0·67 (0·62–0·72)	0·97 (0·95–0·98)	NA	NA
Alcohol	0·75 (0·71–0·78)	0·96 (0·94–0·97)	0·91 (0·89–0·93)	0·87 (0·85–0·90)
Opioid	0·85 (0·81–0·88)	0·99 (0·98–0·99)	0·88 (0·85–0·90)	0·90 (0·87–0·92)
Non-opioid	0·43 (0·35–0·51)	0·96 (0·94–0·97)	NA	NA
**Auxiliary task multilabel convolutional neural network**				
Overall	0·68 (0·63–0·73)	0·97 (0·97–0·98)	NA	NA
Alcohol	0·79 (0·75–0·82)	0·96 (0·95–0·97)	0·92 (0·90–0·94)	0·89 (0·87–0·91)
Opioid	0·87 (0·83–0·90)	0·99 (0·99–0·99)	0·92 (0·90–0·94)	0·95 (0·93–0·96)
Non-opioid	0·38 (0·31–0·46)	0·97 (0·96–0·98)	NA	NA

Temporal validation occurred at Rush University Medical Center. External validation occurred at Loyola University Medical Center. AUPRC=under the precision-recall curve. AUROC=area under the receiver operating characteristic curve. CUI=concept unique identifiers. NA=not applicable.

* Previously published baseline model for classifying alcohol misuse in the hospital.^22^

† Previously published baseline model for classifying opioid misuse in the hospital.^17^

**Table 3:** Bias report for temporal validation dataset (year 2020)

	N	Substance misuse prevalence	False-discovery rate (95% CI)	False-positive rate (95% CI)	False-omission rate (95% CI)	False-negative rate (95% CI)
All encounters	16917	1023	0·25 (0·23–0·29)	0·02 (0·02–0·02)	0·01 (0·01–0·01)	0·18 (0·15–0·20)
Age group, years						
18–44	5336	433	0·19 (0·16–0·23)	0·02 (0·01–0·02)	0·01 (0·01–0·02)	0·16 (0·13–0·20)
≥45	11 581	590	0·30 (0·26–0·33)	0·02 (0·02–0·02)	0·01 (0·01–0·01)	0·18 (0·15–0·21)
Sex						
Female	9944	315	0·29 (0·24–0·34)	0·01 (0·01–0·01)	0·01 (0·01–0·01)	0·22 (0·17–0·27)
Male	6973	708	0·25 (0·22–0·28)	0·03 (0·03–0·04)	0·02 (0·02–0·02)	0·16 (0·13–0·19)
Race and ethnicity						
Non-Hispanic Black	6190	445	0·29 (0·26–0·34)	0·03 (0·02–0·03)	0·03 (0·02–0·03)	0·18 (0·15–0·22)
Non-Hispanic White	6156	353	0·22 (0·18–0·26)	0·01 (0·01–0·02)	0·01 (0·01–0·02)	0·19 (0·15–0·23)
Hispanic	3329	159	0·20 (0·14–0·26)	0·01 (0·01–0·02)	0·01 (0·01–0·02)	0·15 (0·09–0·21)
*Mixed	1242	66	0·32 (0·22–0·43)	0·02 (0·02–0·03)	0·02 (0·02–0·03)	0·14 (0·06–0·24)

Substance misuse prevalence represents any occurrence of alcohol misuse, opioid misuse, or non-opioid drug misuse. The referent labels and predicted labels for any type of substance misuse were used to calculate the number of false positives (FP), true positives (TP), false negatives (FN), and true negatives (TN). Bias assessment metrics included: false-discovery rate (FP/[FP+TP]); false-positive rate (FP/[FP+TN]); false-omission rate (FN/[FN+TN]); and false-negative rate (FN/[FN+TP]).

* Mixed=Asian, Native-American, or Pacific Islander, other, or refused to answer or answer unknown.

## Data Availability

The raw EHR data are not available upon request due to ethical and legal restrictions imposed by the Rush University Medical Center and Loyola University Chicago Institutional Review Boards. The original data are derived from the institutions’ electronic health records and contain patients’ protected health information. Deidentified data are available from the Rush University and Medical Center and Loyola University Chicago for researchers who meet the criteria for access to confidential data and have a data usage agreement with the health system. Only the final trained model that is fully deidentified with a vocabulary of mapped concept-unique identifiers is open-source and available at: https://github.com/Rush-SubstanceUse-AILab/SMART-AI. Our deidentification approach has been previously described.^17^

## Research in context

### Evidence before this study

Substance misuse or unhealthy substance use is a common problem identified in hospitalised patients associated with poor health outcomes, but it is not prioritised, and frequently unaddressed during routine care, because current approaches for screening with structured diagnostic interviews require additional staffing and effort during clinical care. Important details about substance use are captured in the clinical notes of the electronic health record (EHR) but the data are difficult to mine and analyse. Natural language processing (NLP) and machine learning can be used to train models to identify relevant findings in the notes to automatically screen patients with substance misuse. We searched PubMed from Jan 1, 2000, to Dec 31, 2022, for studies indexed in MEDLINE using the key words and Medical Subject Headings terms, (“Electronic Health Records”[Mesh] AND “Natural Language Processing”[MAJR]) AND (“Analgesics, Opioid”[Mesh] OR “Ethanol”[Mesh] OR “Illicit Drugs”[Mesh] OR “Opioid-Related Disorders/diagnosis”[MAJR] OR “Heroin”[Mesh] OR “Alcoholism/diagnosis”[MAJR] OR “excessive alcohol use” OR “unhealthy alcohol use” OR “substance use” or “unhealthy drug use)”. The search was restricted to studies published in English. We identified three studies using machine learning methods to identify single types of substance misuse using domains of data that are not readily available in the EHR and are not comparable. We did identify two studies that built an NLP information extraction system using a keyword rule-based approach but neither employed machine learning in a design for clinical decision support or screening. A third study we found used NLP with machine learning to identify cases of unhealthy substance use from the EHR but the study was focused in paediatric patients and not hospitalised adults.

### Added value of this study

We trained a convolutional neural network to screen and identify alcohol misuse, opioid misuse, and non-opioid drug misuse with high accuracy using notes collected during clinical care. The screening tool uses methods in NLP to screen hospitalised patients to identify individuals with unhealthy substance use behaviours.

### Implications of all the available evidence

An automated solution using notes collected during clinical care could help improve screening rates for substance misuse in hospitals. The screening tool is open-source and publicly available for testing in real-time to determine its effectiveness.
