# Supplementary material for: Development and multimodal validation of a substance misuse algorithm for referral to treatment using artificial intelligence (SMART-AI): a retrospective deep learning study
Source: Lancet Digit Health. Author manuscript; Available in PMC 2022 Jun 1. (PMC9159760; doi:10.1016/S2589-7500(22)00041-3)
Supplement: 1 [file NIHMS1810680-supplement-1.pdf]

THE LANCET
Digital Health

Supplementary appendix

This appendix formed part of the original submission and has been peer reviewed. We post it as supplied by the authors.

Supplement to: Afshar M, Sharma B, Dligach D, et al. Development and multimodal validation of a substance misuse algorithm for referral to treatment using artificial intelligence (SMART-AI): a retrospective deep learning study. *Lancet Digit Health* 2022; **4:** e426–35.

APPENDIX

Appendix 1. Characteristics between internal and external validation hospital sites

Hospital Characteristics	Rush University Medical Center	Loyola University Medical Center
Adult/Pediatric	Yes/Yes	Yes/Yes
Hospital type	Private	Private
Location and primary community area served	West Chicago:<br>60607, 60608, 60612, 60622, 60623, 60624, 60661	West and Southwest Suburbs:<br>60153, 60402, 60104
Inpatient Race/Ethnicity	NH White: 50.4%<br>NH Black: 35.6%<br>Hispanic White: 0.3%<br>Hispanic Black: 1.3%<br>Other/Unknown: 0.2%	NH White: 61.1%<br>NH Black: 21.6%<br>Hispanic White: 5.3%<br>Hispanic Black: 0.1%<br>Other/Unknown: 11.9%
Authorized Beds, n	727	547
EHR System (year deployed)	Epic (2010)	Epic (2007)
Inpatient Insurance	Inpatients:<br>Medicare: 37.2%<br>Medicaid: 22.9%<br>Private insurance: 35.3%<br>Private Pay: 2.3%<br>Charity Care: 2.2%	Inpatients┼<br>Medicare: 41.7%<br>Medicaid: 21.9%<br>Private insurance: 33.1%<br>Private pay: 0.9%<br>Charity care: 2.4%
Emergency Room Visits	66,152	41,123

Appendix 2. TRIPOD Checklist: Prediction Model Development and Validation

Section/Topic	Item		Checklist Item	Page
**Title and abstract**				
Title	1	D;V	Identify the study as developing and/or validating a multivariable prediction model, the target population, and the outcome to be predicted.	1
Abstract	2	D;V	Provide a summary of objectives, study design, setting, participants, sample size, predictors, outcome, statistical analysis, results, and conclusions.	4-5
**Introduction**				
Background and objectives	3a	D;V	Explain the medical context (including whether diagnostic or prognostic) and rationale for developing or validating the multivariable prediction model, including references to existing models.	8-9
	3b	D;V	Specify the objectives, including whether the study describes the development or validation of the model or both.	9
**Methods**				
Source of data	4a	D;V	Describe the study design or source of data (e.g., randomized trial, cohort, or registry data), separately for the development and validation data sets, if applicable.	10,12
	4b	D;V	Specify the key study dates, including start of accrual; end of accrual; and, if applicable, end of follow-up.	FIG. 1
Participants	5a	D;V	Specify key elements of the study setting (e.g., primary care, secondary care, general population) including number and location of centres.	10
	5b	D;V	Describe eligibility criteria for participants.	11
	5c	D;V	Give details of treatments received, if relevant.	N/A
Outcome	6a	D;V	Clearly define the outcome that is predicted by the prediction model, including how and when assessed.	14-15
	6b	D;V	Report any actions to blind assessment of the outcome to be predicted.	12-13
Predictors	7a	D;V	Clearly define all predictors used in developing or validating the multivariable prediction model, including how and when they were measured.	12-13
	7b	D;V	Report any actions to blind assessment of predictors for the outcome and other predictors.	15
Sample size	8	D;V	Explain how the study size was arrived at.	14
Missing data	9	D;V	Describe how missing data were handled (e.g., complete-case analysis, single imputation, multiple imputation) with details of any imputation method.	16, App 8
Statistical analysis methods	10a	D	Describe how predictors were handled in the analyses.	13, App 3-6
	10b	D	Specify type of model, all model-building procedures (including any predictor selection), and method for internal validation.	13-14
	10c	V	For validation, describe how the predictions were calculated.	14-15
	10d	D;V	Specify all measures used to assess model performance and, if relevant, to compare multiple models.	10-11
	10e	V	Describe any model updating (e.g., recalibration) arising from the validation, if done.	15
Risk groups	11	D;V	Provide details on how risk groups were created, if done.	10-12

Development vs. validation	12	V	For validation, identify any differences from the development data in setting, eligibility criteria, outcome, and predictors.	App 1, 9
**Results**				
Participants	13a	D;V	Describe the flow of participants through the study, including the number of participants with and without the outcome and, if applicable, a summary of the follow-up time. A diagram may be helpful.	Fig 1.
	13b	D;V	Describe the characteristics of the participants (basic demographics, clinical features, available predictors), including the number of participants with missing data for predictors and outcome.	Table 1
	13c	V	For validation, show a comparison with the development data of the distribution of important variables (demographics, predictors and outcome).	App 9
Model development	14a	D	Specify the number of participants and outcome events in each analysis.	Fig 1.
	14b	D	If done, report the unadjusted association between each candidate predictor and outcome.	19, App 13
Model specification	15a	D	Present the full prediction model to allow predictions for individuals (i.e., all regression coefficients, and model intercept or baseline survival at a given time point).	19, App 13
	15b	D	Explain how to the use the prediction model.	18
Model performance	16	D;V	Report performance measures (with CIs) for the prediction model.	17
Model-updating	17	V	If done, report the results from any model updating (i.e., model specification, model performance).	18
**Discussion**				
Limitations	18	D;V	Discuss any limitations of the study (such as nonrepresentative sample, few events per predictor, missing data).	24
Interpretation	19a	V	For validation, discuss the results with reference to performance in the development data, and any other validation data.	20-22
	19b	D;V	Give an overall interpretation of the results, considering objectives, limitations, results from similar studies, and other relevant evidence.	22
Implications	20	D;V	Discuss the potential clinical use of the model and implications for future research.	23
**Other information**				
Supplementary information	21	D;V	Provide information about the availability of supplementary resources, such as study protocol, Web calculator, and data sets.	3
Funding	22	D;V	Give the source of funding and the role of the funders for the present study.	2

*Items relevant only to the development of a prediction model are denoted by D, items relating solely to a validation of a prediction model are denoted by V, and items relating to both are denoted D;V. We recommend using the TRIPOD Checklist in conjunction with the TRIPOD Explanation and Elaboration document.

Appendix 3. Approaches for Multi-Label with and without Auxiliary Task Learning

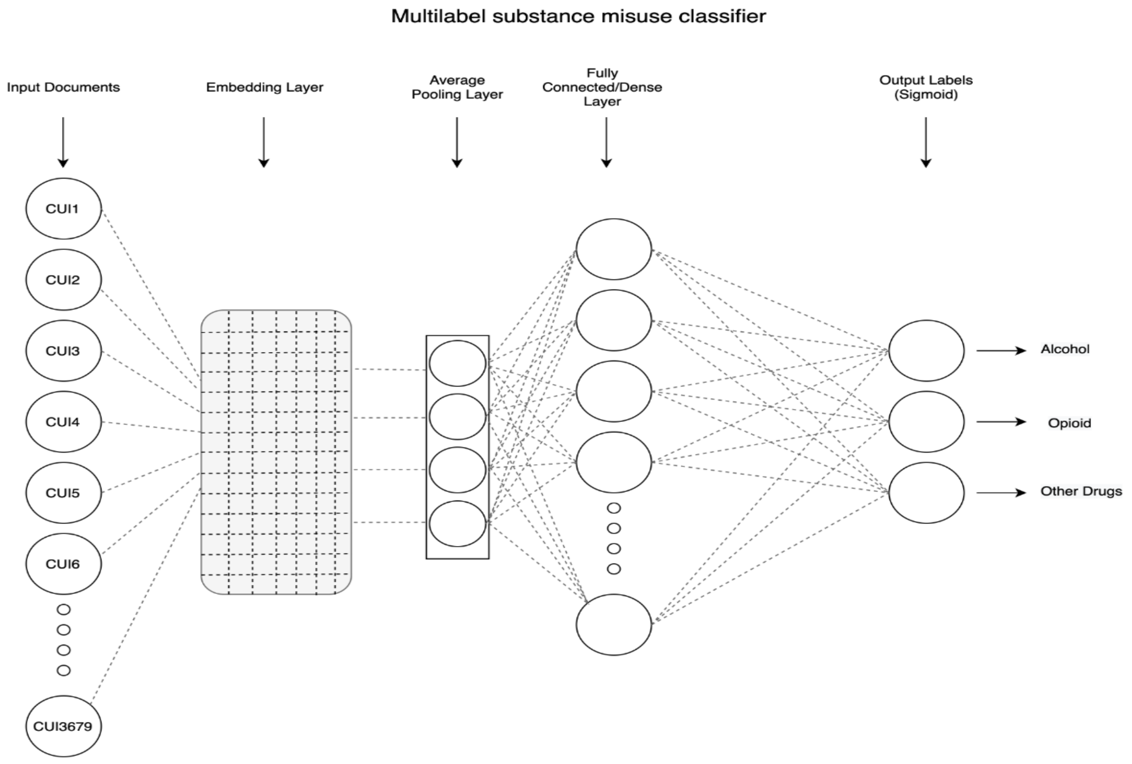

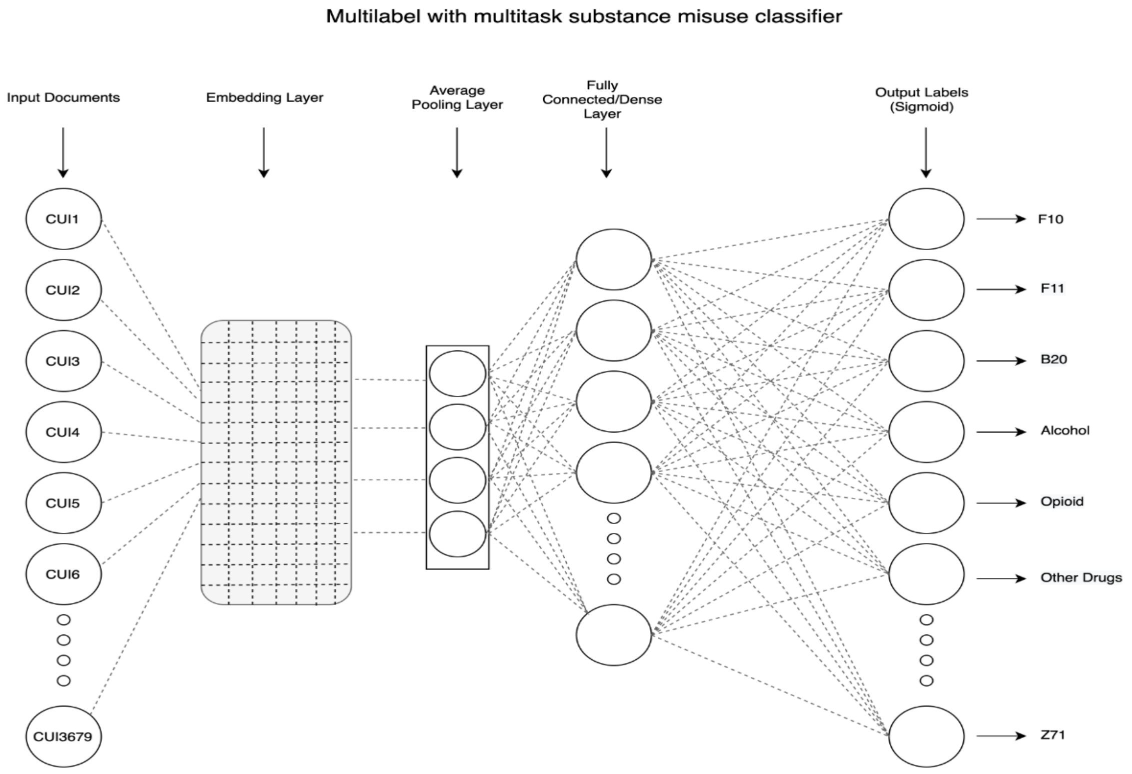

Appendix 4. ICD-10 codes used for Auxiliary Training

**Elixhauser Comorbidity Index**			**Substance User Disorder related ICD-10 codes**		
**Disease**	**ICD 10 Code**	**Count**	**Substance Use Disorder**	**ICD 10 Code**	**Count**
OBESITY	E66.x	19113	Alcohol	F10.x	3414
Hypertension	I10.x	17999			
Fluid and Electrolyte Disorder	E22.2, E86.x, E87.x	16257			
Anemia Deficiency	D50.8, D50.9, D51.x - D53.x	16143	Opioid	F11.x	1895
Hypertension Complication	I11.x-I13.x, I15.x	15549		T40.0x, T40.1x, T40.2x, T40.3x, T40.4x	1510
Diabetes with Chronic Complication	E10.2 - E10.8, E11.2 - E11.8, E12.2 - E12.8, E13.2 - E13.8, E14.2 - E14.8	11775			
Renal Failure	I12.0, I13.1, N18.x, N19.x, N25.0, Z49.0 - Z49.2, Z94.0, Z99.2	11505	Cannabis	F12.x	1895
Chronic Pulmonary Disease	I27.8, I27.9, J40.x - J47.x, J60.x - J67.x, J68.4, J70.1, J70.3	11049			
Weight Loss	E40.x - E46.x, R63.4, R64	10401			
Congestive heart failure	I09.9, I11.0, I13.0, I13.2, I25.5, I42.0, I42.5 - I42.9, I43.x, I50.x, P29.0	10039	Sedative	F13.x	200
Other Neurological Disease	G10.x - G13.x, G20.x - G22.x, G25.4, G25.5, G31.2, G31.8, G31.9, G32.x, G35.x - G37.x, G40.x, G41.x, G93.1, G93.4, R47.0, R56.x	8871			
Depression	F20.4, F31.3 - F31.5, F32.x, F33.x, F34.1, F41.2, F43.2	8294			
Hypothyroidism	E00.x - E03.x, E89.0	6799	Cocaine	F14.x	938

Coagulopathy	D65 - D68.x, D69.1, D69.3 - D69.6	4686			
Valvular Disease	A52.0, I05.x - I08.x, I09.1, I09.8, I34.x - I39.x, Q23.0 - Q23.3, Z95.2 - Z95.4	4320			
Metastatic Cancer	C77.x - C80.x	4281	Other Stimulant	F15.x	84
Peripheral Vascular Disease	I70.x, I71.x, I73.1, I73.8, I73.9, I77.1, I79.0, I79.2, K55.1, K55.8, K55.9, Z95.8, Z95.9	4049			
Solid Tumor without Metastasis	C00.x - C26.x, C30.x - C34.x, C37.x - C41.x, C43.x, C45.x - C58.x, C60.x - C76.x, C97.x	3953			
Liver Disease	B18.x, I85.x, I86.4, I98.2, K70.x, K71.1, K71.3 - K71.5, K71.7, K72.x - K74.x, K76.0, K76.2 - K76.9, Z94.4	3951	Hallucinogen	F16.x	47
Diabetes without Chronic Complications	E10.0, E10.1, E10.9, E11.0, E11.1, E11.9, E12.0, E12.1, E12.9, E13.0, E13.1, E13.9, E14.0, E14.1, E14.9	3873			
Paralysis	G04.1, G11.4, G80.1, G80.2, G81.x, G82.x, G83.0 - G83.4, G83.9	3739			
Rheumatoid Arthritis	L94.0, L94.1, L94.3, M05.x, M06.x, M08.x, M12.0, M12.3, M30.x, M31.0 - M31.3, M32.x - M35.x, M45.x, M46.1, M46.8, M46.9	2721	Inhalant	F18.x	3
Psychoses	F20.x, F22.x - F25.x, F28.x, F29.x, F30.2, F31.2, F31.5	2251			
Alcohol Abuse	F10, E52, G62.1, I42.6, K29.2, K70.0, K70.3, K70.9, T51.x, Z50.2, Z71.4, Z72.1	2070			

Drug Abuse	F11.x - F16.x, F18.x, F19.x, Z71.5, Z72.2	1955	Psychoactive & Other	F19.x	377
Blood Loss Anemia	D50.0	1837			
Pulmonary Circulation Disorder	I26.x, I27.x, I28.0, I28.8, I28.9	1513			
Lymphoma	C81.x - C85.x, C88.x, C96.x, C90.0, C90.2	1359	Non-Psychoactive	F55.x	1
Peptic Ulcer Disease	K25.7, K25.9, K26.7, K26.9, K27.7, K27.9, K28.7, K28.9	1319			
HIV/AIDS	B20.x - B22.x, B24.x	434			

‘x’ here denotes a number between 0-9 if they exist for the ICD code

Appendix 5. Methods for each architecture used

1) Multi-label learning

The model trains on mutually exclusive labels with independent outcomes for alcohol misuse, opioid misuse, and non-opioid drug misuse. We did not provide any weights to the training loss for each misuse type.

2) Multi-task multi-label learning

The model trains on mutually exclusive labels with the independent outcomes for alcohol misuse, opioid misuse, and non-opioid drug misuse, along with substance use disorder-related ICD-9 and -10 codes as secondary labels [Figure]. In total, we had thirty different elixhauser comorbidity indexes, nine different SUD-related ICD code categories. We ran hcuppy python library to gather the elixhauser indexes for each encounter. We consider these extra labels as auxiliary labels intended to provide complexity to the model and improve the model learning capacity for the actual labels. We added weights for the loss as a hyperparameter during the model training. If Ls is a loss for actual outcomes and La for the auxiliary outputs, the total sum of loss is given by:

Total Loss = weight * Ls + (1 - weight * La)

<u>Model Experiments</u>**:**

1) Logistic Regression
   In logistic regression, the training dataset is fed into the model using a bag of CUIs approach. In this approach, we create a matrix of training datasets where every row is an encounter and columns as unique CUIs (n=37317). Each unique CUI in a training document is counted and normalized across the entire document. We experimented with the model using different penalty values, the inverse of regularization strength C ranging from 0.001 to 1000, and class weight as balanced.

2) Feed Forward Neural Network
   In a feed-forward neural network, the training dataset is fed into the model using a bag of CUIs approach. In this approach, we create a matrix of training datasets where every row is an encounter and columns as unique CUIs (n=37317). Each unique CUI in a training document is counted and normalized across the entire document. The matrix passed through a fully connected or dense layer followed by ReLU for non-lineality. Finally, we add a sigmoid output to predict each substance misuse type. We tested with adam, rmsprop, and adagrad for optimizers, different dropouts between 0.1 to 0.9, and a learning rate from 0.01 to 0.0001.

3) Deep Averaging Neural Network

In a deep averaging neural network, the training dataset is limited to a maximum of 12000 words/CUIs. First, we create an embedding layer of dimension 300, which we average across the layer before sending the information to the dense layer, followed by ReLU for non-linearity. In the final layer, we add a sigmoid output for each substance misuse type. We experimented with adam and rmsprop optimizer, range of dropouts from 0.1 to 0.9 and learning rate from 0.01 to 0.0001. These neural networks had relatively fewer parameters to learn than the feed-forward neural network.

4) Convolutional Neural Network
In a convolutional neural network, the training dataset is also limited to a maximum of 12000 words/CUIs. First, we create an embedding layer of dimension 300, followed by the CNN layer with different filter sizes. The learnable weight in this layer is sharable; hence it can extract features from the embedding layer even with a shallow network. The extracted features are sent through the max-pooling layer, followed by a fully connected layer. In the fully connected layer, 2e experimented with units ranging from 8 to 2048. Again, in the final layer, we add a sigmoid output for each substance misuse type. We also tested with adam optimizer, range of dropouts from 0.1 to 0.9 and learning rate from 0.01 to 0.0001.

5) Transformer based Neural Network
Transformer-based neural networks are trending architecture that researchers in recent NLP breakthroughs have used. Transformer models use the attention mechanism, which provides context to each input sequence. Since the encounters have input sequences of larger than 500 CUIs, hence we avoid positional embeddings. The maximum input sequence was 6000, and we experimented with multiple attention heads and layers. Adam optimizer was used, with a learning rate range from 0.1 to 0.0001.

Appendix 6. Hyperparameters for each model

**Model**		
	**Hyperparameters**	**No of Parameters**
**Multi-Label DAN**	dense layer = 15000 units, dropout=0.3, optimizer = adam, learning rate = 0.0001, max length = 12000, batch size = 64	53,632,511
**Multi-Label BOW**	dense layer = 20000 units, dropout = 0.5, optimizer = adam, learning rate = 0.001, batch size = 64	746,420,003
**Multi-Label Transformer**	d_model: 1024, d_inner: 1024, n_head:4, n_layers:1, d_k:768, d_v:96, dropout:0.1, learning rate= 0.0001, batch =32, max length = 6000	47,396,867
**Multi-Label CNN**	embedding size=300, filtersize = 3, filters = 1024, dropout=0.4, dense layer = 32 units, adam, learning rate = 0.0001, batch size = 128**,** max length = 12000	12,521,156
**Auxiliary-Task Multi-Label DAN**	embedding size = 1024, dense dayer = 20000 units, dropout=0.4, adam, learning rate = 0.001, weight = 0.5, batch size = 64, max length = 12000	54,217,650
**Auxiliary-Task Multi-Label BOW**	dense layer: 15000 units, dropout= 0.5, learning rate: 0.001, optimizer = adam, weight = 0.5, batch size = 64	560,400,042
**Auxiliary-Task Multi-Label Transformer**	d_model: 1024, d_inner: 1024, n_head:4, n_layers:1, d_k:768, d_v:96, dropout:0.1, learning rate= 0.0001, max length=6000, weight= 0.9, batch: 128	47,436,842
**Auxiliary-Task Multi-Label CNN**	embedding size = 300, weight = 0.8, filtersize = 3, filters = 1024, dropout=0.5, dense = 32, optimizer = adam, learning rate = 0.0001, batch size = 128, max length = 12000	12,151,910

We split the training data set into 90% training set (n = 49423) and 10% development set (n = 5492). We trained using the training set and the model selection using the development set. We used a random search approach to tune the hyperparameters across each of the models. In random search, hyperparameters are selected randomly from a pool of hyperparameter space. The process repeats several times until we find the best hyperparameters that give the highest pr auc score. Unlike grid search, we did not use every combination of parameters from the hyperparameter space for a random search.

Hence, the parameter search time is quicker and still yields the best result. We experimented with eight different experiments using four Tesla V100 GPUs, python 3.6, PyTorch 1.4 version. For each of these experiments, we ran a random search until we found the best precision-recall area under the curve.
BOW = bag of words, DAN = deep averaging network, CNN = convolutional neural network, d_model = the number of expected feature in encoder/decoder input, d_k = keys for dimension, d_v = values for dimension, n_head = number of heads in the multi attention layer, max length = maximum length of the document, dense layer = number of neurons

Appendix 7. eXplainable Artificial Intelligence (XAI) with Local Interpretable Model-agnostic Explanations (LIME)

We applied the LIME package to understand the highest weighted features that discriminate between cases and non-cases. LIME uses a local approximation to interpret a black box model such as neural networks by developing a linear model and assigning weights to each feature. For model selection, a grid search approach was applied to a small training dataset (30 documents) to get the best average R^2 value to find the best hyperparameters. The primary hyperparameters we experimented with were feature selection (“forward selection”, “auto” “lasso_path”, and “none”) and kernel width (ranging from 1 to 7). Then, we ran LIME on the 2000 subset of the entire training dataset keeping the prevalence of substance misuse the same as that of the whole cohort. The weights for all the features (n = 37371) for each document were averaged and sorted to produce the top 25 weighted features. Next, we repeated the experiment for all the misuse types. For each misuse type, the best hyperparameter selected was “auto” for feature selection and 2 for the kernel width. The R^2 values for alcohol, opioid, and nonopioid are 0.951, 0.961, 0.962, respectively.

Appendix 8. Not screened or incomplete screening data vs screened data characteristics

	**Not screened or Incomplete Screening (n=25715)**	**Screened (n=60567)**	**p - value**
Age, median (IQR)	62.0 (45.0 - 74.0)	60.0 (43.0, 71.0)	<0.0001
Male sex, n (%)	10,643 (41.4%)	25953 (42.8%)	<0.0001
Race+Ethnicity, n (%)			<0.0001
Non-Hispanic White	11205 (43.5%)	25725 (42.5%)	
Non-Hispanic Black	8978 (34.9%)	19850 (32.8%)	
Hispanic/Latinx	3349 (13.0%)	10594 (17.5%)	
Mixed race+ethnicity	2183 (8.5%)	4398 (7.3%)	
Insurance, n (%)			<0.0001
Medicare	9542 (37.1%)	21199 (35.0%)	
Medicaid	8253 (32.1%)	20459 (33.8%)	
Private	6655 (25.9%)	16024 (26.5%)	
Other	1265 (4.9%)	2885 (4.8%)	
Comorbidities:			
Hypertension, n (%)	13488 (52.5%)	36220 (59.8%)	<0.0001
Renal Failure, n (%)	4956 (19.3%)	12080 (19.9%)	0.023
Neurologic, n (%)	4029 (15.7%)	9681 (15.9%)	0.249
Congestive heart failure, n (%)	4400 (17.1%)	10624 (17.5%)	0.129
Diabetes, n (%)	6426 (24.9%)	16577 (27.4%)	<0.0001
Liver disease, n(%)	1109 (4.3%)	4405 (7.3%)	<0.0001
Chronic lung disease, n(%)	4791 (18.6%)	12033 (19.9%)	<0.0001
Psychiatric disorders, n(%)	1563 (6.1%)	2778 (4.6%)	<0.0001
Depression, n(%)	3073 (11.9%)	9193 (15.2%)	<0.0001
Alcohol Misuse, n(%)	995 (3.9%)	2882 (4.8%)	<0.0001

Drug Misuse, n(%)	934 (3.6%)	2637 (4.4%)	<0.0001
AIDS, n(%)	166 (<1%)	498 (<1%)	<0.0001
Discharge Disposition (n%)<br><br>Home<br>In-Hospital Death<br>LT RC / ST PA<br>AMA<br>Other	 <br><br>13236 (51.5%)<br>1739 (6.7%)<br>3271(9.2%)<br>272 (3.6%)<br>7197(14.6%)	 <br><br>35676 (58.9%)<br>744 (<1%)<br>7999 (13.2%)<br>604 (<1%)<br>15544 (25.7%)	<0.0001

AMA = against medical advice; AIDS = acquired immunodeficiency syndrome; LT RC/ST PA = Long term residential care or short term post acute care

Appendix Table 9. Patient Characteristics and Outcomes from Training Cohort (n = 54915)

	**Alcohol Misuse Only (n=927)**	**Opioid Misuse Only (n=579)**	**Non-opioid Misuse Only (n=195)**	**Polysubstance Misuse (n=220)**	**No Misuse (n=52994)**
Age, median (IQR)	49.0 (39.0 – 59.0)	49.0 (38.0 – 58.0)	52.0 (37.5 – 60.0)	45.0 (32.0 - 56.0)	61.0 (45.0 - 72.0)
Male sex, n (%)	621 (66.9%)	368 (63.6%)	108(55.4%)	142 (64.5%)	21370(40.3%)
Race+Ethnicity,n(%)					
NH White	400 (43.1%)	177 (30.6%)	27 (13.8%)	74 (33.6%)	22367(42.2%)
NH Black	263 (28.4%)	314 (54.2%)	122(62.6%)	97 (44.1%)	17284(32.6%)
Hispanic	201 (21.7%)	67 (11.6%)	35 (17.9%)	35 (15.9%)	9446(17.8%)
Mixed	63 (6.8%)	21 (1.1%)	11 (5.6%)	14 (6.4%)	3897 (7.4%)
AUDIT Score, median (IQR)	19.0 (11.0, 28.0) (n = 927)	0.0 (0.0, 1.0) (n = 128)	2.0 (0.0, 4.0) (n = 60)	21.0 (13.0, 29.0) (n=220)	2.0 (0.0, 3.0) (n = 1989)
DAST Score, median (IQR)	1.0 (0.0, 1.0) (n = 175)	6.0 (4.0, 8.0) (n = 579)	3.0 (2.0, 6.0) (n = 195)	5.0 (3.0, 7.0) (n = 220)	0.0 (0.0, 1.0) (n = 1363)
Insurance, n (%)					
Medicare	133 (14.3%)	73 (12.6%)	31 (15.9%)	14 (6.4%)	19872(37.5%)
Medicaid	432 (46.6%)	405 (69.9%)	117(60.0%)	133 (60.4%)	17056(32.2%)
Private	232 (25.0%)	69 (11.9%)	30 (15.4%)	33 (15.0%)	13832(26.1%)
Other	130 (14.0%)	32 (5.5%)	17 (8.7%)	40 (18.2%)	2234(4.2%)
Comorbidities, n(%)					
Hypertension	512 (55.2%)	291 (50.3%)	128(65.6%)	96 (43.6%)	32441(61.2%)
Renal Failure	83 (8.9%)	89 (15.4%)	51 (26.2%)	15 (6.8%)	11243(21.2%)
Neurologic	206 (22.2%)	86 (14.9%)	29 (14.9%)	45 (20.5%)	8478(15.9%)
CHF	104 (11.2%)	111 (19.2%)	63 (32.3%)	19 (8.6%)	9722(18.3%)
Diabetes	137 (14.8%)	100 (17.3%)	56 (28.7%)	34 (15.5%)	15291(28.9%)
Liver disease	318 (34.3%)	78 (13.5%)	14 (7.2%)	42 (19.1%)	3489(6.6%)
Chronic lung disease	174 (18.8%)	222 (38.3%)	74 (37.9%)	58 (26.4%)	10498(19.8%)

Psychiatric disorders	82 (8.8%)	99 (17.1%)	57 (29.2%)	43 (19.5%)	1967 (3.7%)
Depression	259 (27.9%)	137 (23.7%)	43 (22.1%)	61 (27.7%)	7779 (14.7%)
Alcohol Misuse	742 (80.0%)	56 (9.7%)	36 (18.5%)	168 (76.4%)	1061 (2.0%)
Drug Misuse	98 (10.6%)	560 (96.7%)	153(78.5%)	168 (76.4%)	971 (1.8%)
AIDS	22 (2.4%)	20 (3.5%)	16 (8.2%)	9 (<1%)	367 (<1%)
Disposition, n (%)					
Home	623 (67.2%)	327 (56.5%)	113(57.9%)	138 (62.7%)	30770(58.1%)
Death	7 (<1%)	2 (<1%)	1 (<1%)	2 (<1%)	597 (1.1%)
LT RC / ST PA	120 (12.9%)	109 (18.8%)	27(13.8%)	33 (15.0%)	7216 (13.6%)
AMA	24 (2.6%)	56 (9.7%)	9 (4.6%)	20 (9.0%)	335 (<1%)
Other	153 (16.5%)	85 (14.7%)	45(23.1%)	27 (12.3%)	1407626.6(%)

Polysubstance misuse can include patients with alcohol and/or opioid misuse and/or non-opioid drug misuse; AMA = against medical advice; AIDS = acquired immunodeficiency syndrome; LT RC/ST PA = Long term residential care or short term post acute care; CHF = congestive heart failure; NH= non-Hispanic; Mixed = Asian, Native American or Alaskan Native, Native Hawaiian or Other Pacific Islander, Other, or Refuse/Unknown

Appendix 10. Full List of Cutpoints and Confusion Matrix for Temporal Validation Cohort for multi-label CNN classsifier

Misuse Type	F1	PPV	Sens	NPV	Spec	Cutpoint	Confusion Matrix
Alcohol	0.7	0.63	0.78	0.99	0.98	0.02	452, 125, 264, 16074
Opioid	0.76	0.65	0.91	1	0.99	0.02	354, 33, 193, 16337
Other Drug	0.45	0.33	0.72	1	0.99	0.02	123, 48, 247, 16499
Alcohol	0.73	0.73	0.74	0.99	0.99	0.05	425, 152, 159, 16181
Opioid	0.8	0.73	0.89	1	0.99	0.05	343, 44, 130, 16400
Other Drug	0.46	0.41	0.52	1	0.99	0.05	89, 82, 129, 16617
Alcohol	0.74	0.79	0.69	0.99	0.99	0.1	400, 177, 109, 16231
Opioid	0.81	0.77	0.86	1	0.99	0.1	332, 55, 100, 16430
Other Drug	0.43	0.47	0.4	0.99	1	0.1	68, 103, 77, 16669
Alcohol	0.74	0.83	0.67	0.99	1	0.15	384, 193, 81, 16259
Opioid	0.81	0.79	0.84	1	0.99	0.15	326, 61, 87, 16443
Other Drug	0.38	0.5	0.31	0.99	1	0.15	53, 118, 54, 16692
Alcohol	0.74	0.85	0.65	0.99	1	0.2	375, 202, 68, 16272
Opioid	0.81	0.8	0.83	1	1	0.2	320, 67, 79, 16451
Other Drug	0.34	0.52	0.25	0.99	1	0.2	43, 128, 40, 16706
Alcohol	0.72	0.86	0.62	0.99	1	0.25	355, 222, 57, 16283
Opioid	0.82	0.82	0.82	1	1	0.25	318, 69, 71, 16459
Other Drug	0.32	0.57	0.22	0.99	1	0.25	38, 133, 29, 16717
Alcohol	0.71	0.87	0.6	0.99	1	0.3	347, 230, 51, 16289
Opioid	0.82	0.84	0.8	1	1	0.3	310, 77, 61, 16469
Other Drug	0.3	0.6	0.2	0.99	1	0.3	34, 137, 23, 16723
Alcohol	0.7	0.88	0.59	0.99	1	0.35	339, 238, 46, 16294
Opioid	0.82	0.85	0.79	1	1	0.35	306, 81, 54, 16476

Other Drug	0.26	0.59	0.17	0.99	1	0.35	29, 142, 20, 16726
Alcohol	0.7	0.9	0.57	0.99	1	0.4	329, 248, 38, 16302
Opioid	0.82	0.86	0.78	0.99	1	0.4	302, 85, 50, 16480
Other Drug	0.25	0.65	0.15	0.99	1	0.4	26, 145, 14, 16732
Alcohol	0.69	0.9	0.55	0.98	1	0.45	319, 258, 34, 16306
Opioid	0.82	0.87	0.77	0.99	1	0.45	299, 88, 45, 16485
Other Drug	0.23	0.69	0.14	0.99	1	0.45	24, 147, 11, 16735
Alcohol	0.67	0.91	0.53	0.98	1	0.5	304, 273, 31, 16309
Opioid	0.82	0.88	0.77	0.99	1	0.5	297, 90, 40, 16490
Other Drug	0.2	0.71	0.12	0.99	1	0.5	20, 151, 8, 16738
Alcohol	0.74	0.84	0.66	0.99	1	0.174	383, 194, 74, 16266
Opioid	0.82	0.89	0.76	0.99	1	0.528	295, 92, 36, 16494
Opioid Drug	0.22	0.73	0.13	0.99	1	0.463	22, 149, 8, 16738

Appendix 11. Single default Cutpoint and Confusion Matrix for External Validation Cohort for multi-label CNN classifier

**Type**	F1	PPV	Sens	NPV	Spec	Cutpoint	Confusion Matrix
Opioid	0.55	0.98	0.38	0.76	1	0.5	130, 210, 2, 650
Alcohol	0.64	0.95	0.49	0.58	0.96	0.5	283, 297, 15, 404

Appendix 12. Calibration Plots

Alcohol Non-Calibrated:

Unreliability Index P value: <0.01

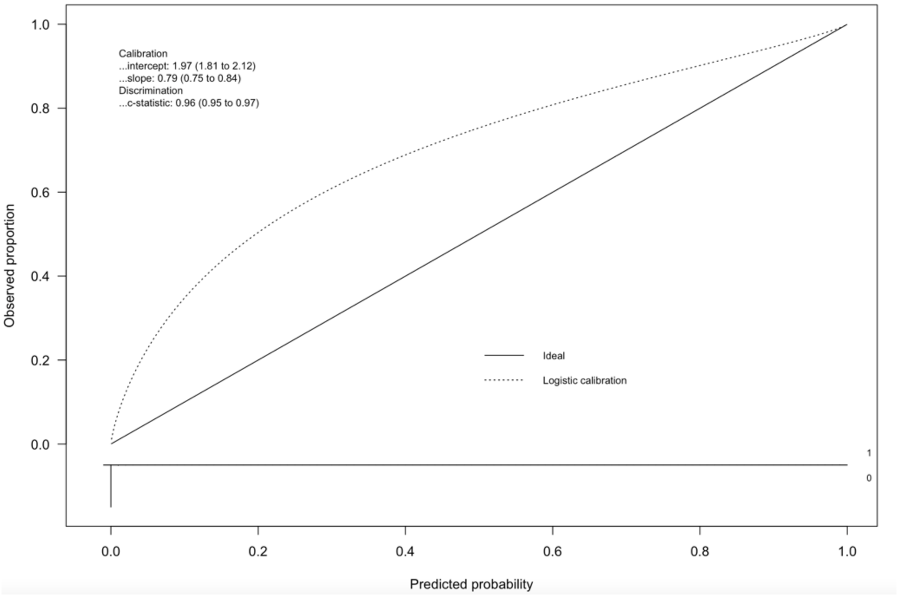

Alcohol Calibrated:

Unreliability Index P value: 0.135

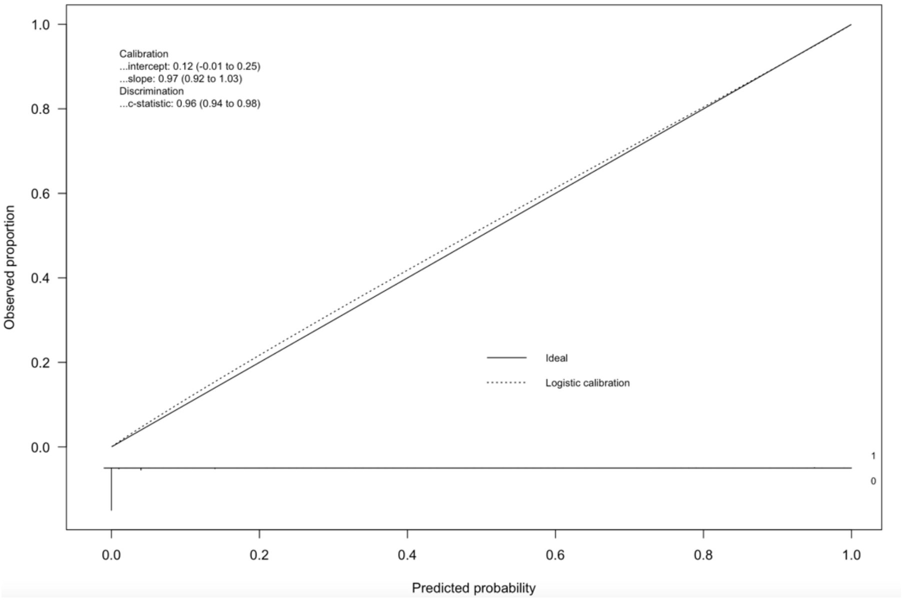

Opioid Non-Calibrated:

Unreliability Index P value <0.0001

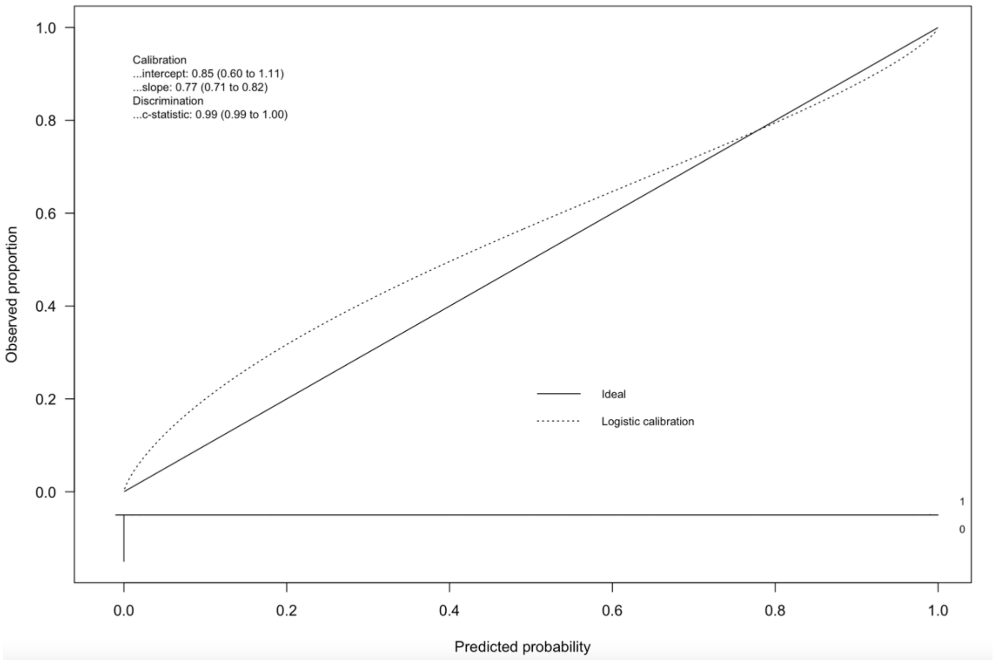

Opioid Calibrated:

Unreliability Index P value: 0.095

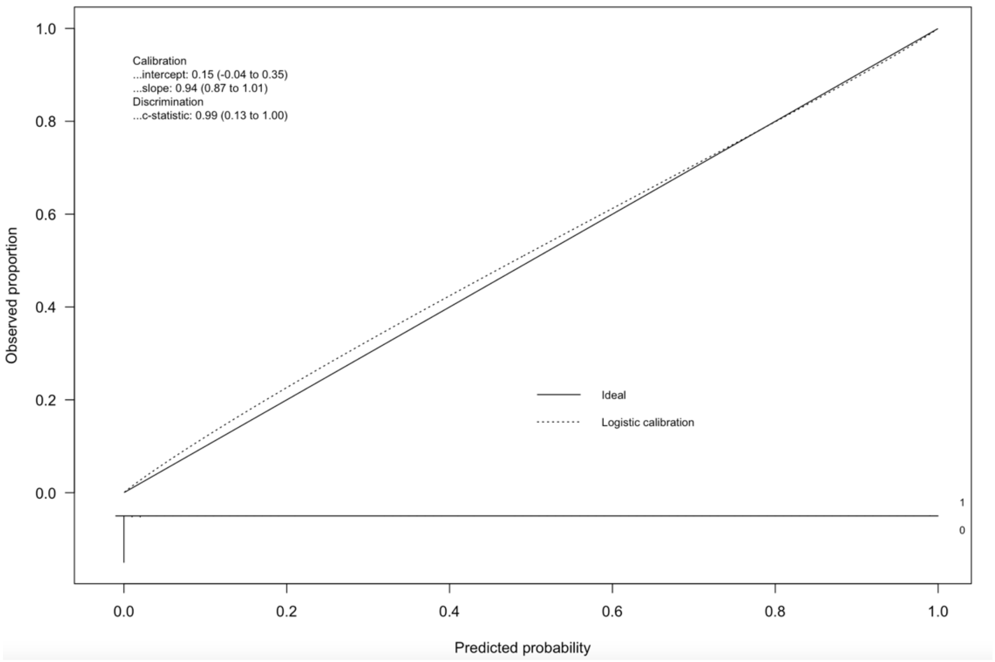

Non-Opioid Non-Calibrated:

Unreliability Index P value: 0.01

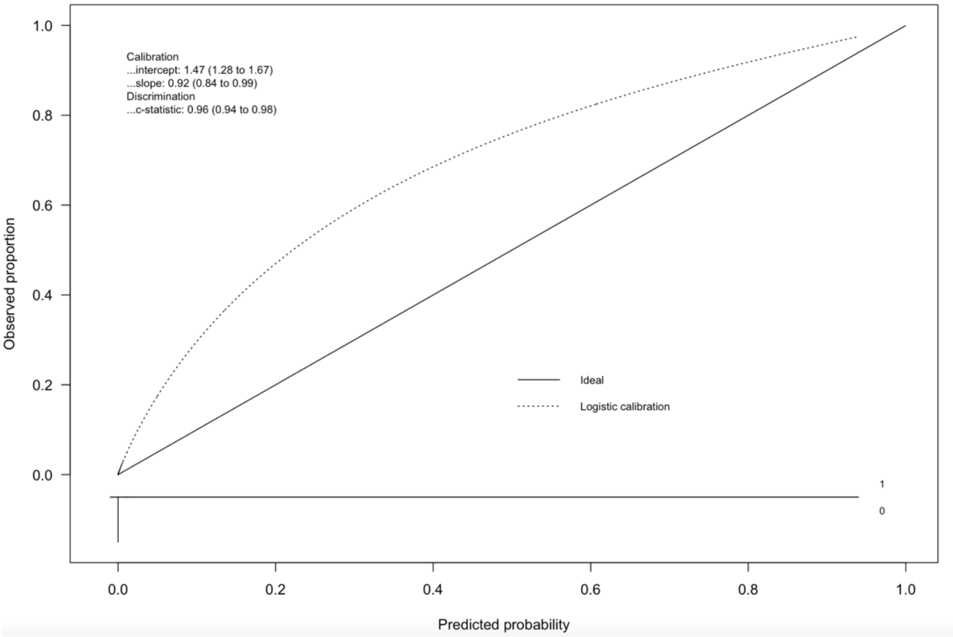

Non-Opioid Calibrated:

Unreliability Index P value: 0.051

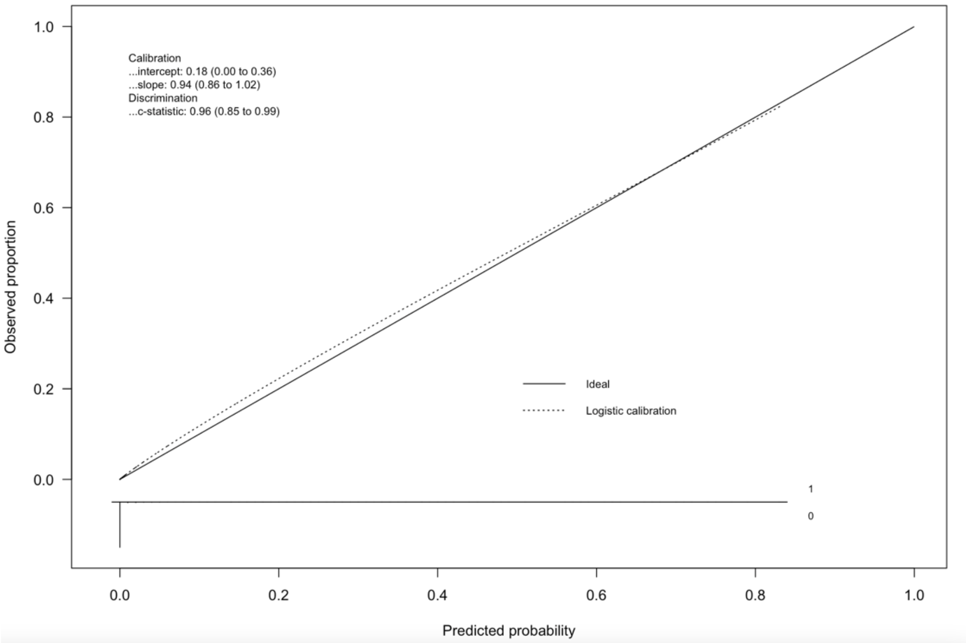

Appendix 13. Top 25 terms from a vocabulary of 37,317 unique terms

Alcohol Misuse		Opioid Misuse		Non-Opioid Drug Misuse	
**CUI text**	**Weight**	**CUI text**	**Weight**	**CUI text**	**Weight**
Ethanol	0.0058	Heroin	0.0046	Cocaine	0.0042
Alcohol Abuse	0.0010	Smokes tobacco daily	0.0007	Victim of abuse finding	0.0013
Thiamine	0.0010	Cocaine	0.0006	Cocaine user	0.0011
Drink (dietary substance)	0.0010	Victim of abuse finding	0.0005	Drug abuse	0.0010
Victim of abuse finding	0.0009	Opioids	0.0004	Cocaine Abuse	0.0007
Beer	0.0005	Methadone	0.0004	Smokes tobacco daily	0.0006
Cocaine	0.0005	Drug abuse	0.0004	Substance	0.0004
Yes - Presence findings	0.0004	Suboxone	0.0003	Polysubstance abuse	0.0004
Vitamins	0.0004	Opiates	0.0003	Marijuana	0.0003
Smokes tobacco daily	0.0004	Heroin Dependence	0.0002	Ethanol	0.0003
Alcohol withdrawal syndrome	0.0004	Substance	0.0002	Screening for cancer	0.0003
Diazepam	0.0003	Cocaine user	0.0001	Drink (dietary substance)	0.0002
Vitamin B complex	0.0003	Tobacco user	0.0001	Positive	0.0002
Valium	0.0002	Chest	0.0001	Substance abuse problem	0.0002
Drinking function	0.0002	Polysubstance abuse	0.0001	Crack Cocaine	0.0002
Disease	0.0002	Blood culture	0.0001	Substance Abuse Detection	0.0001
Multivitamin preparation	0.0002	Possible	0.0001	Beer	0.0001
Vomiting	0.0002	Substance abuse problem	0.0001	Disruptive, Impulse Control, and Conduct Disorders	0.0001
Complication	0.0002	Positive	0.0001	Type II Mucolipidosis	0.0001
Carbon Dioxide	0.0002	Pain	0.0001	Injection procedure	0.0001
Distilled Alcoholic Beverage	0.0002	Asthma	0.0001	Tobacco user	0.0001
Drug abuse	0.0002	Thoracic aorta	0.0001	Pain	0.0001
Screening for cancer	0.0002	Social and personal history	0.0001	Scleral icterus	0.0001
Ativan	0.0002	PMH- past medical history	0.0001	Mean Corpuscular Volume	0.0001
Vodka	0.0002	Sweating	0.0001	Joints	0.0001

A sampling of 2,000 patients from the training cohort of 54,915 hospitalizations between 2017 and 2019. The features represent the related text for the Concept Unique Identifier (CUI) from the National Library of Medicine Metathesaurus
